# Prevalence and risk factors of hypovitaminosis D in pregnant Spanish women

**DOI:** 10.1038/s41598-020-71980-1

**Published:** 2020-09-25

**Authors:** Andrés Díaz-López, Cristina Jardí, Marcela Villalobos, Nuria Serrat, Josep Basora, Victoria Arija

**Affiliations:** 1 Medicine and Health Sciences Faculty, Universitat Rovira i Virgili, Reus, Spain; 2 Institut d’Investigació Sanitària Pere Virgili (IISPV), Reus, Spain; 3 Centro de Investigación Biomédica en Red Fisiopatología de la Obesidad y la Nutrición (CIBEROBN), Institute of Health Carlos III, Madrid, Spain; 4 Research Group in Nutrition and Mental Health (NUTRISAM), Universitat Rovira i Virgili, Reus, Spain; 5 Laboratory ICS Tarragona, University Hospital of Tarragona Joan XXIII, Catalan Institute of Health (ICS), Tarragona, Spain; 6 Unit of Research Support Reus-Tarragona, Institut d´Investigació en Atenció Primària, IDIAP Jordi Gol, Tarragona, Spain

**Keywords:** Health care, Medical research, Risk factors

## Abstract

The hypovitaminosis D epidemic is a global health problem. Our aim was to assess the prevalence and potential risk factors of hypovitaminosis D among pregnant women on the eastern Mediterranean coast. Cross-sectional analysis involved 793 healthy pregnant women (35.3 ± 5.0 years) participating in ECLIPSES, a multicenter randomized trial. Socio-demographic, obstetric, anthropometric, lifestyle, dietary variables and blood draw was collected in the first trimester. Vitamin D deficiency was identified in 50.2% and insufficiency in 30.3% of pregnant women. The mean vitamin D level in the overall sample was 33.9 nmol/L (SD, 17.0). Multivariable logistic regression analysis applying AIC-based backward selection identified excess weight during the 1st trimester (BMI ≥ 25 kg/m^2^) (OR = 1.950, 95% CI = 1.409, 2.699), Arab ethnic group/dark skin colour (OR = 4.005, 95% CI = 2.488, 6.447), winter/spring (OR = 4.319, 95% CI = 3.112, 5.994), and consumption of milk (OR = 0.754, 95% CI = 0.572, 0.993) and yogurt (OR = 0.635, 95% CI = 0.436, 0.922) as independent risk factors for vitamin D deficiency. All of these factors (except yogurt consumption) and physical activity were independently associated with vitamin D deficiency/insufficiency risk in the final multivariable model (all *p* < 0.05). All these factors and social class were the most important determinants of circulating 25(OH)D concentrations. Our results confirm a high prevalence of vitamin D deficiency and insufficiency among pregnant women from the eastern Mediterranean coast.

## Introduction

It is well established that vitamin D deficiency is a worldwide public health problem of epidemic proportions in all age groups^1^. Current global estimates indicate that vitamin D deficiency affects 1 billion people in the world and that women of reproductive age and pregnant women are at high risk of developing vitamin D deficiency^1,2^. Vitamin D deficiency in pregnancy has been associated with maternal health problems such as preeclampsia^3^ and gestational diabetes^4,5^. In addition, adverse outcomes on the newborn, including lower birth weight^4^, height, and cephalic circumference^6^ and higher risk of small for gestational age^7^, or preterm infants^8^ have been related to vitamin D deficiency in pregnancy.

In this regard, numerous epidemiological studies conducted in Europe, including the Mediterranean regions, have described a high prevalence of hypovitaminosis D among pregnant women, although the prevalence rates reported varies across studies (ranging from 1 to 78%)^9–19^. Indeed, a direct comparison between studies is not possible due to wide variations in geographical location, study design, study population, and the cutoff values used to define vitamin D deficiency and insufficiency. Surprisingly, despite Spain being one of Europe's sunniest countries, the few studies available show a relatively high prevalence of vitamin D deficiency among pregnant women^12,13,15^. This may be attributed, at least in part, by less time spent outdoors, low skin exposure due to clothing choices, the use of sunscreens, or avoiding exposure to UVB-radiation^14^. In addition, other factors such as blood sampling in winter^12,20^, excess weight^14^, non-European ethnicity, physical inactivity, low socio-economic and education levels, or low dietary intake of foods containing vitamin D could contribute to vitamin D deficiency in pregnant women^14,21^. However, the impact of each risk factor on vitamin D deficiency/insufficiency in pregnant women varies among European countries and Mediterranean regions with different dietary and lifestyle habits or non-modifiable (genetics) factors^10,11^. Therefore, determining the mother’s vitamin D status in early pregnancy and associated factors is essential if strategies are to be developed for preventing/controlling this serious pandemic health problem and guiding future public health policies.

According to evidence, vitamin D supplements have a positive impact on vitamin D status in pregnant women and a potential role in the prevention of hypovitaminosis D during pregnancy^22,23^. However, there is still a need for additional robust research trials on the beneficial effects that vitamin D supplements taken during pregnancy have on maternal or neonatal outcomes. Whereas the World Health Organization (WHO) does not recommend vitamin D supplementation for pregnant women to improve maternal and perinatal outcomes, it does encourage women to receive adequate nutrition through the consumption of a healthy and balanced diet^24^.

To date, epidemiological studies on maternal vitamin D status in early pregnancy and its determinants in healthy pregnant women living in Spain, especially on the Mediterranean coast (with generally high and rates of sunshine) are scarce^11,15^ and further research is required. Thus, the objective of this study was to assess the prevalence of vitamin D deficiency and deficiency/insufficiency in the first trimester of gestation and to identify potential risk factors of hypovitaminosis D in pregnant Spanish women on the eastern coast of the Mediterranean. This means increasing our knowledge on the vitamin D status of pregnant women in the Spain population and may contribute significantly to the public health and clinical area, because those pregnant women who are at high risk of vitamin D deficiency could be screened or intervened in advance to prevent the deficiency during pregnancy.

## Materials and methods

### Study population

A cross-sectional analysis was conducted in the framework of the ECLIPSES trial. Details of the study's protocol have been described elsewhere^25^. Briefly, ECLIPSES was a community randomized controlled trial (RCT) conducted in the province of Tarragona (Catalonia, Spain) between 2013 and 2017 that aimed to assess maternal health status during pregnancy (considering nutritional, psychological and environmental factors) and its association with offspring outcomes (including physical and neurobehavioral development)^25^. A total of 793 women were recruited during the first prenatal visit (before week 12 of pregnancy) from 12 sexual and reproductive health care services (ASSIR) of the Catalan Institute of Health (ICS) in Tarragona, Spain. The ECLIPSES trial was registered in the EU Clinical Trial Register, EUCTR-2012-005480-28 and in ClinicalTrials.gov with identification number NCT03196882. This study was approved by the Ethical Committee of the Institut d'Investigació en Atenció Primaria de Salut (IDIAP) and the Institut d'Investigació Sanitària Pere Virgili (IISPV). All participants signed an informed consent form. Eligible participants were healthy adult women older than 18 years with ≤ 12 weeks of gestation. Further details of the inclusion/exclusion criteria can be found elsewhere^25^.

### Study variables

Information about maternal age, ethnicity, socio-economic level, education level, estimated date of delivery, risk factors during pregnancy, family planning, use of contraceptives, clinical history, obstetric data, toxic habits (smoking, consumption of alcohol and drugs), blood pressure (the average of three recordings), height (cm) and weight (kg) was obtained by interview using validated questionnaires. Vitamin D supplementation was recorded from the periconceptional period up to 12 months. On the basis of the criteria proposed by the World Health Organization (WHO), BMI was classified as normal weight (BMI < 25 kg/m^2^) and excess weight (BMI ≥ 25 kg/m^2^)^26^. Dietary intake was assessed using a validated semiquantitative food frequency questionnaire (FFQ) interview-administered to pregnant women at 12 weeks^27^. The FFQ consisted of 45 items classified into 12 food groups. Participants specified their usual food consumption in common portions or serving sizes retrospectively. From this information, we extracted the consumption of fatty fish (servings [1 serving = 100 g]/week), milk (servings [1 serving = 220 g]/day), yogurt (servings [1 serving = 125 g]/day), and dietary vitamin D intake (μg/day) (not including vitamin D intake from dietary supplements). The average consumption rations were compared with the dietary guidelines of the Sociedad Española de Nutrición Comunitaria (SENC)^28^. The vitamin D content of the food items was primarily estimated using the official French food composition table^29^ and other tables published for Spanish foods^30^. To evaluate adequacy, the intake of vitamin D was compared with the recommended intake values of the Institute of Medicine of the National Academies of the United States and Canada^31^. Physical activity (PA) was measured using the short version of the International Physical Activity Questionnaire (IPAQ-S)^32^. The type of PA (walking, moderate intensity and vigorous intensity), frequency (number of times a week) and duration (min/day) were obtained^33^. In addition, to obtain the metabolic equivalents (MET) in minutes per week for each type of PA, the usual frequency and duration were averaged (min/week) and multiplied by a constant according to their energy expenditure (walking: 3.3 MET; moderate intensity: 4.0 MET; vigorous intensity: 8,0 MET) and MET-min/week were obtained. Total PA was obtained by summing the MET-min/week for each type of PA.

### Assessment of circulating 25(OH)D concentration

Blood was extracted from the pregnant women before week 12 of gestation. Samples were processed immediately and stored at − 80 °C in the BioBank of the reference hospital until analysis. Serum concentrations of 25(OH)D total (i.e. comprising the sum of 25(OH)D_2_ and 25(OH)D_3_) were quantified by an automated chemiluminescent immunoassay method (ADVIA Centaur VitD). According to the manufacturer’s specifications, the intra-assay coefficient of 398 variation was of 4.2–11.9% and functional sensitivity was of 3.3 ng/mL (8.3 nmol/L). Measurement interval was 4–150 ng/mL. The analytical specificity reflected through the percentage of cross-reactivity with other metabolites was 97.4% for 25(OH)D_3_, 106.2% for 25(OH)D_2_ and 1% for C_3_ epimer 25(OH)D_3_. We defined vitamin D deficiency, insufficiency, and sufficiency as serum 25(OH)D concentrations of < 30 nmol/L (< 12 ng/mL), 30–50 nmol/L (12–20 ng/mL), and ≥ 50 nmol/L (≥ 20 ng/mL), respectively, as recommended by the Institute of Medicine^31^.

### Statistical analysis

Statistical analysis was performed using the STATA software, version 15.0 (StataCorp LLC, TX. USA). Quantitative variables were expressed as mean and standard deviation (SD) and qualitative variables as numbers and percentages (%). Differences between groups were examined by the independent Student's t test, χ^2^ or ANOVA as appropriate. Univariable and multivariable logistic regression analyses were used to assess risk factors related to vitamin D deficiency (compared with vitamin D non-deficiency) or deficiency/insufficiency (compared with vitamin D sufficiency) in pregnancy and presented as odds ratios (OR) and 95% confidence intervals (CIs). For multivariable logistic regression, the initial model included all factors that showed significance after a likelihood ratio test Chi-square test of the univariable logistic regression and was further refined by Akaike information criterion (AIC)-based backward selection (STATA "swaic, model back" command). Potential confounders in the initial multivariable model included: age (years), BMI (normal weight [BMI < 25 kg/m^2^], excess weight [BMI ≥ 25 kg/m^2^] [reference]), ethnicity (white [reference], non-white [Arab ethnic origin, dark skin colour]), education level (primary studies, secondary studies, university studies [reference]), socio-economic level (high [reference], low/middle), smoking habit (no [reference], yes [smoker or ex smoker]), PA during 1st trimester (METS-min/week), season of the year at blood collection (summer/fall [reference], winter/spring), milk intake during 1st trimester (servings/day), and yogurt intake during 1st trimester (servings/day). Age was considered as a clinically relevant variable and was included in multivariable models. Before modelling, we evaluated the multicollinearity of factors embedded in the multivariable model by the general variance inflation factor. There was none. The goodness-of-fit of the final model was tested by the Hosmer–Lemeshow statistic and Nagelkerke R^2^.

Additionally, multivariable linear regression analysis was performed to identify the leading determinants of serum 25(OH)D concentrations in the first trimester by calculating the regression coefficients (β) and associated 95% CI. All factors associated with vitamin D deficiency by the univariable logistic regression were considered for inclusion in the multivariable model and an AIC stepwise backwards selection was used to arrive at the final model. Statistical significance was set at *p* < 0.05.

### Ethical approval

All procedures performed in the study were in accordance with the ethical standards of the Institut d'Investigació en Atenció Primaria de Salut (IDIAP) and the Institut d'Investigació Sanitària Pere Virgili (IISPV) and with the 1964 Helsinki declaration and its later amendments or comparable ethical standards.

## Results

A total of 793 pregnant women (mean age 30.5 ± 5.0 years) were enrolled in the study. Participants were categorised as having deficient (< 30 nmol/L), insufficient (30–50 nmol/L), or sufficient (≥ 50 nmol/L) vitamin D status. The results of the participants’ general characteristics according to vitamin D status are shown in Table 1. We observed significant differences between 25(OH)D groups in terms of maternal BMI during first trimester, ethnicity, education level, socioeconomic status, smoking habit, PA during first trimester, and season in which blood was collected. Regarding the intake of vitamin D-rich foods during the first trimester, women with deficient or insufficient levels of vitamin D were less likely to consume dairy products, specifically milk and yogurt (Table 1). No other differences were identified between 25(OH)D groups (Table 1).

**Table 1:** General characteristics of pregnant women in the total sample and in terms of vitamin D status in the first trimester of pregnancy.

Characteristics	N	Mean (SD), nmol/L	25(OH)D categories, n (%)			
			Deficiency < 30 nmol/L N = 398 (50.2)	Insufficiency 30–50 nmol/L N = 240 (30.3)	Sufficiency ≥ 50 nmol/L N = 155 (19.5)	*p*^‡^
Serum 25(OH)D concentration (nmol/L), mean (SD)	793	33.9 (17.1)	20.3 (5.6)†	38.7 (5.5)#	61.6 (9.1)	**< 0.001**
Age (years), mean (SD)	793	30.5 (5.0)	30.4 (5.0)	31.1 (5.0)	30.3 (4.9)	0.159
**BMI (kg/m**^**2**^**) during 1st trimester, mean (SD)**						
Normal weight (BMI < 25 kg/m^2^)	500	36.1 (7.8)	219 (55.0)†	167 (69.6)	114 (74.2)	**< 0.001**
Excess weight (BMI ≥ 25 kg/m^2^)	293	30.2 (14.9)*	179 (44.5)	73 (30.4)	41 (25.8)	
**Ethnicity**						
White	667	35.9 (17.1)	301 (75.6)†	217 (90.4)#	149 (96.1)	**< 0.001**
Non-white (Arab or dark skin)	126	23.0 (11.9)*	97 (14.6)	23 (1.3)	6 (3.9)	
**Education level**						
Primary studies	249	31.2 (16.7)	148 (37.2)†	61 (25.4)	40 (25.8)	**0.009**
Secondary studies	324	34.6 (17.3)	153 (38.4)	101 (42.1)	70 (45.2)	
University studies	220	35.9 (16.8)*	97 (24.4)	78 (32.5)	45 (29.0)	
**Social class**						
Low/medium	663	32.9 (16.7)	347 (87.2)†	193 (80.4)	123 (79.3)	**0.023**
High	130	39.0 (18.2)*	51 (12.8)	47 (19.6)	32 (20.6)	
**Smoking habit**						
No	579	32.6 (16.8)	309 (77.6)†	167 (69.6)	103 (66.4)	**0.010**
Yes (smoker or ex-smoker)	214	37.3 (17.4)*	89 (22.4)	72 (30.4)	52 (33.6)	
Physical activity during 1st trimester (METS-min/week), mean (SD)	793	689.8 (880.5)	623.7 (837.1)†	689 (789.5)	860.5 (1,082.7)	**0.018**
**Parity**						
No	297	34.8 (17.6)	142 (35.7)	94 (39.2)	61 (39.4)	0.584
Yes	496	33.4 (16.7)	256 (64.3)	146 (60.8)	94 (60.6)	
**Season at blood collection**						
Winter/spring	491	28.7 (14.1)	308 (77.4)†	137 (57.1)#	46 (29.7)	**< 0.001**
Summer/fall	302	42.5 (17.9)*	90 (22.6)	103 (42.9)	109 (70.3)	
**Alcohol consumption during 1st trimester**						
No	789	33.9 (17.1)	395 (99.2)	239 (99.6)	155 (100)	0.518
Yes	4	25.3 (16.6)	3 (0.8)	1 (0.4)	0 (0.0)	
Vitamin D intake from diet during 1st trimester (µg/day), mean (SD)	793	1.79 (0.93)	1.78 (0.94)	1.81 (1.0)	1.77 (0.81)	0.932
Fatty fish intake during 1st trimester (servings/ week), mean (SD)	793	1.28 (1.08)	1.27 (1.10)	1.29 (1.13)	1.29 (0.96)	0.979
Milk intake during 1st trimester (servings/day), mean (SD)	793	0.97 (0.59)	0.92 (0.56)†	0.98 (0.62)#	1.10 (0.57)	**0.005**
Yogurt intake during 1st trimester (servings/day), mean (SD)	793	0.52 (0.42)	0.49 (0.40)†	0.57 (0.43)	0.57 (0.44)	**0.025**

The significance of numbers in bold is *p*-value < 0.05.

Values are expressed in means (SD, standard deviation) or number (%). BMI, Body mass index; METS, Metabolic equivalents.*Statistically significant differences in serum 25(OH)D concentrations for intragroup comparisons at *p* < 0.05 as derived from Student's t/ANOVA tests, as appropriate. ^‡^*p* values for the differences between 25(OH)D categories (deficiency, insufficiency or sufficiency) as derived from ANOVA or χ^2^ tests, as appropriate. *p* < 0.05 for the differences between †deficiency versus sufficiency and #insufficiency versus sufficiency.

The mean serum 25(OH)D concentrations in the overall sample was 33.9 nmol/L (SD, 17.0). Of the total number of pregnant women in their first trimester participating in our study, 50.2% (n = 398) had vitamin D deficiency, 30.3% (n = 240) had insufficiency, and 19.5% (n = 155) of women presented sufficient levels of vitamin D. Mean 25(OH)D levels among pregnant women differed significantly depending on maternal BMI, ethnicity, education level, social class, smoking habit, and season in which blood was collected (Table 1). Serum 25(OH)D concentrations did not differ significantly between the first and the third trimesters (33.6 nmol/L, *p* = 0.636). Of note, the overall prevalence of vitamin D deficiency (49.72%, n = 394), insufficiency (33.2%, n = 263), or sufficiency (17.2%, n = 136) was similar at the third trimester (data not shown).

Univariable logistic regression analysis showed that excess weight during the 1st trimester (BMI ≥ 25 kg/m^2^) (OR = 2.015, 95% CI = 1.502, 2.703; *p ≤ *0.001), Arab ethnic group/dark skin colour (OR = 4.067, 95% CI = 2.614, 6.327; *p ≤ *0.001), low/medium social class (OR = 1.701, 95% CI = 1.159, 2.496; *p* = 0.007), and season in which blood was collected (winter/spring) (OR = 3.964, 95% CI = 2.916, 5.389; *p ≤ *0.001) were factors associated with a risk of vitamin D deficiency. Conversely, a lower risk of vitamin D deficiency was significantly associated with a high education level (OR = 0.538, 95% CI = 0.373, 0.777; *p* = 0.001), smoking (OR = 0.622, 95% CI = 0.453, 0.854; *p* = 0.003), PA (OR = 0.999, 95% CI = 0.999, 0.999; *p* = 0.037), and consumption of milk (OR = 0.732, 95% CI = 0.571, 0.939; *p* = 0.014) and yogurt (OR = 0.626, 95% CI = 0.444, 0.882; *p* = 0.008) during the first trimester. The factors that had a statistically significant association with vitamin D deficiency, apart from social class, education level, and yogurt consumption, were also significantly associated with vitamin D deficiency/insufficiency (all *p* < 0.05) (Table 2). All these probable factors plus age were analyzed by AIC-based multivariable stepwise backward logistic regression models to identify independent risk factors for vitamin D deficiency or deficiency/insufficiency. After multivariable analysis, excess weight (OR = 1.950, 95% CI = 1.409, 2.699; *p ≤ *0.001), Arab ethnic group/dark skin colour (OR = 4.005, 95% CI = 2.488, 6.447; *p ≤ *0.001), blood collection in winter/spring (OR = 4.319, 95% CI = 3.112, 5.994; *p ≤ *0.001), and consumption of milk (OR = 0.754, 95% CI = 0.572, 0.993; *p* = 0.045) and yogurt (OR = 0.635, 95% CI = 0.436, 0.922; *p* = 0.017) remained strong predictors of risk of vitamin D deficiency. All these factors (except yogurt consumption) and PA were independently associated with the risk of vitamin D deficiency/insufficiency in the final multivariable model (all *p* < 0.05). The goodness-of-fit of the final model was assessed to be appropriate by the Hosmer–Lemeshow statistic and Nagelkerke R^2^. All other possible factors were not associated with vitamin D deficiency or deficiency/insufficiency in the final model (Table 2).

**Table 2:** Results of uni- and multivariable logistic regression models of factors associated with vitamin D deficiency (< 30 nmol/L) or deficiency/insufficiency (< 50 nmol/L) in the first trimester of pregnancy (n = 793).

	Vitamin D deficiency				Vitamin D deficiency/insufficiency			
	Univariable models		Multivariable model*		Univariable models		Multivariable model*	
	OR (95% CI)	*p*	OR (95% CI)	*p*	OR (95% CI)	*p*	OR (95% CI)	*p*
Age (years)	0.983 (0.956, 1.011)	0.235			1.013 (0.978, 1.049)	0.471		
BMI (Kg/m^2^) during 1st trimester (Excess weight, BMI ≥ 25 kg/m^2^)	2.015 (1.502, 2.703)	**< 0.001**	1.950 (1.409, 2.699)	**< 0.001**	1.815 (1.228, 2.683)	**0.003**	1.696 (1.107, 2.600)	**0.015**
Ethnicity (Arab or dark skin)	4.067 (2.614, 6.327)	**< 0.001**	4.005 (2.488, 6.447)	**< 0.001**	5.753 (2.483, 13.324)	**< 0.001**	5.691 (2.389, 13.556)	**0.030**
Education level (secondary studies)	0.611 (0.437, 0.853)	**0.004**			1.433 (0.919, 2.236)	0.096		
Education level (university studies)	0.538 (0.373, 0.777)	**0.001**			0.744 (0.464, 1.191)	0.219		
Social class (low/ medium)	1.701 (1.159, 2.496)	**0.007**			1.433 (0.919, 2.235)	0.112		
Smoking habit (yes)	0.622 (0.453, 0.854)	**0.003**			0.674 (0.462, 0.984)	**0.041**		
Physical activity	0.999 (0.999, 0.999)	**0.037**	0.999 (0.999, 1.000)	0.073	0.999 (0.999, 0.999)	**0.009**	0.999 (0.999, 0.999)	**0.022**
Parity (yes)	1.164 (0.873, 1.552)	0.300			1.105 (0.771, 1.585)	0.586		
Season at blood collection (winter/spring)	3.964 (2.916, 5.389)	**< 0.001**	4.319 (3.112, 5.994)	**< 0.001**	5.463 (3.722, 8.019)	**< 0.001**	5.825 (3.906, 8.687)	**< 0.001**
Vitamin D intake from diet during 1st trimester (µg/day)	0.983 (0.846, 1.141)	0.823			1.014 (0.839, 1.226)	0.880		
Fatty fish intake during 1st trimester (servings/ week)	0.986 (0.867, 1.121)	0.836			0.991 (0.843, 1.164)	0.913		
Milk intake during 1st trimester (servings/day)	0.732 (0.571, 0.939)	**0.014**	0.754 (0.572, 0.993)	**0.045**	0.657 (0.495, 0.871)	**0.004**	0.616 (0.449, 0.847)	**0.003**
Yogurt intake during 1st trimester (servings/day)	0.626 (0.444, 0.882)	**0.008**	0.635 (0.436, 0.922)	**0.017**	0.752 (0.507, 1.116)	0.157		
Goodness-of-fit			Nagelkerke R^2^ = 25.1%; H–L statistic = 9.15, DF = 8, *p* = 0.330				Nagelkerke R^2^ = 24.6%; H–L statistic = 5.59, DF = 8, *p* = 0.693	

The significance of numbers in bold is *p*-value < 0.05.

BMI, Body mass index; METS, Metabolic equivalents; OR, Odds Ratio; CI, confidence intervals; H–L, Hosmer–Lemeshow; DF = degree of freedom. *Model obtained by an AIC-based backward selection from all statistical variables in the univariable analysis.

Vitamin D insufficiency (compared with vitamin D sufficiency) had a similar pattern of results to vitamin D deficiency. In a multivariate analysis of 395 women and with AIC-based backward selection, age (OR = 1.046, 95% CI = 1.001, 1.093), Arab ethnic group/dark skin colour (OR = 3.043, 95% CI = 1.160, 7.980), winter/spring (OR = 3.320, 95% CI = 2.135, 5.162), and milk consumption (OR = 0.654, 95% CI = 0.454, 0.942) were independently associated with vitamin D insufficiency in the first trimester. (OR = 0.754, 95% CI = 0.572, 0.993). The Hosmer–Lemeshow goodness-of-fit test showed that the model fitted the data well (*p* = 0.781).

Furthermore, we constructed a corroborative multivariable linear regression analysis to explore the determinants of serum 25(OH)D concentrations in the first trimester using the same set of possible factors associated with vitamin D deficiency at the univariate analysis. The best model, shown in Table 3, explained 29% of the variance in serum 25(OH)D concentrations. In this model, BMI group, ethnicity, social class, PA, season at sampling, and consumption of milk and yogurt during the first trimester were independent determinants of 25(OH)D concentrations.

**Table 3:** Results of multivariable linear regression analysis of determinants of circulating 25(OH)D concentration (nmol/L) in the first trimester of pregnancy (n = 793).

Determinants	β	SE	(95% CI)	*p*
Age (years)	− 0.161	0.109	− 0.376, 0.0532	0.138
BMI (Kg/m^2^) during 1st trimester (Excess weight, BMI ≥ 25 kg/m^2^)	− 4.320	1.097	− 6.474, − 2.156	**< 0.001**
Ethnicity (Arab or dark skin)	− 10.334	1.518	− 13.316, − 7.353	**< 0.001**
Social class (low/ medium)	− 3.667	1.484	− 6.579, − 0.753	**0.014**
Smoking habit (yes)	2.114	1.218	− 0.277, 4.506	0.083
Physical activity during 1st trimester (METS-min/week)	0.001	0.001	0.0002, 0.002	**0.022**
Season at blood collection (winter/spring)	− 13.246	1.080	− 15.367, − 11.126	**< 0.001**
Milk intake during 1st trimester (servings/day)	1.947	0.904	0.173, 3.721	**0.031**
Yogurt intake during 1st trimester (servings/day)	3.014	1.258	0.544, 5.485	**0.017**

The significance of numbers in bold is *p*-value < 0.05.

β, coefficient of regression CI, confidence intervals; SE, standard error.

Nagelkerke R^2^ = 26.6%; F_9, 778_ = 31.51; *p* < 0.001.

Other variables such as age, parity, vitamin D intake from diet and fatty fish intake during the first trimester were not significantly related to 25(OH)D levels. None of our participants were on vitamin D supplementation.

## Discussion

Our study confirms that healthy pregnant Spanish women, despite living on the Mediterranean coast with abundant sunshine all year round even in winter (Tarragona), have a high prevalence of both vitamin D deficiency (50.2%) and insufficiency (30.3%) in the first trimester of pregnancy. We also found that the risk factors associated with lower vitamin D status during the first trimester of gestation were excess weight, Arab ethnic origin or dark skin colour, blood collection in winter/spring, PA, and consumption of dairy products, specifically milk and yogurt. All these factors plus social class were shown in our study to be the most important determinants of circulating 25(OH)D concentrations.

The prevalence of both vitamin D deficiency and insufficiency among pregnant women in the first trimester of pregnancy is higher than that observed in other European countries and other parts of Spain. Previous epidemiological studies conducted in northern European countries, where the population is considered to have a high risk of vitamin D deficiency due to less exposure to solar UVB, revealed that the prevalence of vitamin D deficiency ranged from 1 to 26%^9,34–37^. Perhaps, the policies implemented in northern European countries have had a profound effect on the prevalence of vitamin D deficiency in their population. In Norway for example, it has decreased from 60–70 to 1% among pregnant women^9^. In these studies, at least 40% of pregnant women were taking vitamin D supplements, but in ours no women reported using supplements (only some used iodine, folic acid or vitamin B12 supplements). Although in a study conducted in Spain of three different areas (Valencia, Sabadell and Guipuzkoa at latitudes of 39° N, 41° N and 42° N, respectively), the overall prevalence of vitamin D insufficiency was found to be 18.0%. In particular the prevalence in Sabadell (40.9%), an area near to ours, was quite similar to that observed in our study^12^. And the prevalence of vitamin D insufficiency in Guipuzkoa was indeed higher than in our study (64.4%). It should be noted that only 5.8% of pregnant women were taking vitamin D supplements and the latitude was considerably lower than ours^13^. In addition, previous epidemiological studies in our country also show a high prevalence of hypovitaminosis (32.6% of deficiency and 86.3% of insufficiency) in the general population^38–40^ and this has been linked to little exposure to the sun and a low intake of food rich in vitamin D.

It is well known that skin pigmentation influences the effectiveness of vitamin D synthesis, and ethnicity is an important non-modifiable determinant of circulating 25(OH)D concentrations. We found that Arab ethnic origin and dark skin colour are potential predictors of lower vitamin D status in our population. In a recent multicenter epidemiological study investigating the association of maternal vitamin D concentrations and anthropometric parameters of the child, a higher prevalence of vitamin D deficiency was reported (Vitamin D levels < 50 nmol/L) among women from the Middle East (36%) than in European women (1–7%)^41^. Blarduni et al. reported that women of non-European origin had a higher risk of vitamin D deficiency than other ethnic groups (OR: 13.09)^13^. Women of Arab, African and/or South American origin with darker skin tones have a higher concentration of melanin which may prevent the absorption of UVB rays and, therefore, vitamin D synthesis^42^. In addition, clothing may prevent exposure to the sun and consequently decrease the endogenous synthesis of vitamin D. Supporting this hypothesis, epidemiological studies in Turkey, a country where most of the population is Muslim, found an association between clothing and vitamin D deficiency^43^. It should be noted that in our study we found that women of Arab origin were more likely to be overweight (54.1% *vs*. 33.6%), to spend less time walking outside (251.3 METS for women of Arab origin *vs*. 357.1 METS for white women) and to consume less milk (data not shown), all of which are risk factors for hipovitaminosis D. This could contribute to the higher prevalence of vitamin D deficiency and insufficiency found in this ethnic group.

Another important factor related to lower vitamin D levels is the phenomenon known as seasonal variability. Our results show lower vitamin D levels during the winter/spring months, and a higher risk of vitamin D deficiency and insufficiency, which is in accordance with previous studies conducted in our country^12,13^. The association of BMI with vitamin D status has been analysed in many countries, although the results have been inconsistent^10,12,13,44,45^. We found that pregnant women with excess weight (BMI ≥ 25 kg/m^2^) had lower circulating 25(OH)D and a greater probability of presenting lower vitamin D status. In this regard, it has been hypothesized that the high risk of vitamin D deficiency among overweight and obese individuals is most probably due to excess adiposity, even though inadequate vitamin D intake cannot be excluded, as they may contribute concurrently^46^.

Another important source of vitamin D is dietary intake. In the present study, we did not find an association between dietary vitamin D intake and risk of vitamin D deficiency during early pregnancy. On average, we found that the mean vitamin D intake among pregnant women in the first trimester of pregnancy was 1.8 (1.1) μg/day, and was similar in the 25(OH)D groups. In this respect, previous studies in the Spanish population have reported a daily consumption of vitamin D ranging from 2.9 μg^47^ to 4.4 µg^48^. To our knowledge, only one previous study in Spain has assessed the impact of dietary intake on vitamin D status^12^, and it found that dietary vitamin D intake did not meet the recommendations for pregnant women proposed by the Institute of Medicine in 2011 (15 μg/day)^31^. Our study, conducted in pregnant women who did not take vitamin D supplements, showed that a higher consumption of dairy products (milk and yogurt) was associated with higher levels of vitamin D. In addition, a higher intake of milk decreased the odds of both vitamin D deficiency and insufficiency. However, we did not find any associations between fish intake, a good source of vitamin D, and vitamin D levels, probably due to the low frequency of fish consumption in our population (19.6 g/day).

Social class, education and ethnic origin can be related to the lower intake of some food groups; for this reason, we assessed the intake of fish and milk in different social classes and ethnic groups. Our results showed that women of Arab origin were significantly less likely to consume dairy products than white women. In terms of fish consumption, there were no significant differences for social classes or ethnic groups.

PA was another lifestyle factor identified as significantly impacting the vitamin D status of pregnant women in our study. Previous studies conducted in Spain^12,13^, Indonesia^49^ and Saudi Arabia^50^ have reported that PA in pregnant women could protect against vitamin D deficiency. A very recent randomized clinical trial carried out in pregnant women in Norway shows that PA during pregnancy positively affects the vitamin D levels of the pregnant woman^51^. In fact, the benefits of outdoor PA have been attributed to sun exposure.

In line with previous reports, our study shows a high prevalence of hypovitaminosis D during pregnancy and confirms that vitamin D deficiency is a global epidemic affecting people of all age groups. In view of our results it is important that measuring vitamin D status in pregnant women should be an integral part of health examinations in pregnant women in primary care. In addition, we suggest that health professionals take into account all these risk factors to ensure adequate vitamin D status through physical activity and the consumption of dairy products during pregnancy and to prevent adverse outcomes in offspring. In particular, they should consider the most vulnerable groups: women of Arab origin or dark skin colour, women of low social class and low education level, pregnancy during winter/spring and excess weight. Although in Spain vitamin D fortification is not widespread, a number of vitamin D-fortified milk products are currently available^52^. One potential strategy for improving vitamin D status in the general population in our country would be to systematically fortify dairy products, such as milk and yogurt. In this regard, previous studies demonstrate that vitamin D-fortified milk products have a positive impact on vitamin D status^53^. Even so, more research is needed to explore the safety of vitamin D fortification, including the range of products and the doses of vitamin D added. Furthermore, studies of vitamin D supplementation in pregnancy are needed to determine the effects of vitamin D supplementation in pregnancy and to identify the optimal dose for supplementation.

The present study has some strengths that need to be mentioned. It provides current information on the prevalence of vitamin D deficiency and insufficiency among Spanish pregnant women in the Mediterranean area. The sample is relatively large, and contains different ethnic groups, social classes, and levels of education. Our results may be applicable to other groups of pregnant women. It also provides information on a large number of the risk factors that may impact on vitamin D status in pregnancy. Both dietary data and physical activity data were collected by using validated questionnaires, which increase the validity of our findings. It is well known that the normal range of 25(OH)D varies somewhat depending on the measurement method used. Unlike other analytical methods from published studies, the ADVIA Centaur Vitamin D Total assay used in our study is traceable to the Ghent University ID-LC–MS/MS 25(OH)D reference measurement procedure for vitamin D testing. Furthermore, it has been demonstrated that the 25(OH)D results in serum samples from pregnant women obtained by the ADVIA Centaur Vitamin D Total immunoassay were equivalent to the sum of 25(OH)D2 and 25(OH)D3 using the LC–MS/MS method^54^.

One of the limitations of the study is that we were not able to determine the number of hours that women spent outdoors, whether they used sunscreens, how much skin was exposed, or if they were exposed to particulate air pollution. In addition, its cross-sectional design does not allow us to establish a cause–effect relationship, and the possibility of reverse causation should be acknowledged. Another limitation was not evaluated other periods of gestation, focusing on the 1st trimester, since previous manuscripts indicate that vitamin D levels are maintained throughout pregnancy and even decrease. In our study, serum 25(OH)D concentrations did not differ significantly depending on trimesters.

## Conclusions

In conclusion, a high prevalence of vitamin D deficiency and insufficiency has been detected among healthy pregnant women on the Mediterranean's eastern coast. The factors most related to lower levels of vitamin D levels in pregnant women were excess weight, Arab ethnic group and dark skin, low social class, little exposure to sunlight and less efficient dermal vitamin D synthesis during the winter and spring, low consumption of dairy products, and low physical activity. We suggest including vitamin D assessments in existing antenatal care settings in order to control and prevent vitamin D deficiency and the associated consequences, while considering high-risk groups.
